# Predictors of Residential Mobility among Older Canadians and Impact on Analyses of Place and Health Relationships

**DOI:** 10.3934/publichealth.2015.1.115

**Published:** 2015-03-24

**Authors:** Mathieu Philibert, Mark Daniel

**Affiliations:** 1 Département de sexologie, Université du Québec à Montréal, Montréal, Canada; 2 Institut national de santé publique du Québec, Montréal, Canada; 3 Spatial Epidemiology and Evaluation Research Group, School of Population Health, Sansom Institute for Health Research, University of South Australia, Adelaide, Australia; 4 Department of Medicine, The University of Melbourne, St Vincent's Hospital, Melbourne, Australia; 5 South Australian Health and Medical Research Institute, Adelaide, Australia

**Keywords:** residential mobility, neighbourhood, environmental factors, bias

## Abstract

This study aimed to identify predictors of residential mobility in 55+ Canadians, to characterise neighbourhood changes following mobility, to assess whether such changes differ according to income, and to evaluate for cross-sectional estimations of place-health relationships the extent of bias associated with residential mobility. Using longitudinal data from the Canadian National Population Health Study (NPHS), residential mobility was operationalised by a change in postal code between two consecutive waves. Individuals' sociodemographic factors and neighbourhood characteristics were analysed in relation to mobility. Bias in cross-sectional estimations of place-health associations was assessed analysing neighbourhood-level deprivation and housing quality in relation to self-assessed health. Multiple age-related events were predictive of moving. Three out of 10 individuals moved at least once. Two thirds of movers experienced a change in neighbourhood type and such changes were not associated with income. No systematic biases in estimating place effects on health using cross-sectional data were observed. Given that individual-level socioeconomic status (SES) was neither a predictor of moving nor of its consequences in terms of neighbourhood type, controlling for SES could potentially lead to biased estimations of place-health associations. Results suggest that cross-sectional data can yield valid estimations of place-health associations among older adults.

## 1. Introduction

Numerous studies have reported associations between features of the residential environment and individuals' health or health-related behaviours. Health disparities are considered to arise, in part, due to current and historic exposures to environmental influences. Social and built environmental factors are among the many influences affecting health over the life course [1]–[3]. Of health and place studies that have focused specifically on older populations, however, most have limitations, including the use of cross-sectional designs that do not support ascertaining the temporal antecedence of exposures against health outcomes [4],[5].

Associations between health and places characteristics estimated from cross-sectional data preclude accounting for residential mobility in its impact on the estimated associations. Such estimations may be biased if the observed distribution of key residential factors is unrepresentative of the distribution of such influences in their impact on the distribution of health across the life course. In effect, individuals and entire households may relocate from one residential area to another across the life course, such that substantial variations can exist in exposures to health-related residential area factors. Discrepancies between current and past exposures to such factors are all the more important for understanding the role of place effects on health among the aged. This group will by its very nature reflect by extended empirical induction periods over which disease processes can develop based on past residential area exposures and, potentially, the impact of mobility from area to area thus shaping disease outcomes.

Self-selection (i.e., the systematic movement of certain types of individuals to certain types of places and neighbourhoods) has the potential to bias the estimated associations between place and health at a given time if the individual-level characteristics driving residential mobility either include or are associated with health status. This can lead to reverse causation, that is, health status itself driving an observed association with place rather than being the outcome of some feature of a place. Bias from reverse causation in area-level estimates of place effects on health is now thought, however, to have a lesser impact on conclusions drawn from general population studies than was previously thought. The impact of residential mobility in potentially biasing associations estimated for aged populations is unknown, however. It is in this population that any such bias stands to have its greatest impact.

In older adults, residential mobility is often triggered by age-related events including health decline, retirement, change in income, widowhood, and children moving from the parental home to a home of their own [6]–[8]. When driven by health-related factors, mobility can change local population health profiles as well as modify demand in use of local health and social services.

For those who relocate, a frequent outcome of residential mobility is change in one's environment. Such changes can be minor, or substantial. Yet mobility will not always correspond to change in the features of the environments to which individuals are exposed, notably when one moves laterally rather than up or down a vertical scale of exposure. For example, setting aside the primary reason for moving, one's choice of a new residential area is influenced by individual or household financial resources. Many will be constrained to a similar residential neighbourhood. Therefore, residential mobility may not drastically change the nature of key features to which individuals are exposed. At the population level, if lateral moves (between neighbourhoods of similar socioeconomic status, as opposed to vertical moves) represent the greatest frequency of moves, mobility might not modify, or not greatly modify, the population distribution of exposures to key environmental influences.

Little is known about the extent to which residential mobility leads to changes in the distribution of environmental influences relevant to population health. Also unknown is the impact of such changes on place-health relationships estimated from cross-sectional data and, in particular, how residential mobility bears on place-health relationships estimated for aged populations, given that many studies report results broadly for all individuals at or beyond the age of adulthood, or actively exclude older age groups in analyses [e.g., 9].

This longitudinal study sought to determine over time the antecedent predictors and consequences of residential mobility in older Canadians. Several reasons justify a focus on older adults' exposure to residential factors [10]. Firstly, declines in health can increase sensitivity to the influence of environmental stresses and obstacles on disability. Second, in comparison to younger adults, aged populations are more exposed to features of the local residential environment due to greater longevity and movement restrictions. Third, older adults can be more dependent on local resources due to the shrinkage, over time, of the social network by which they access various resources that shape health [11].

This study had four objectives. First, it aimed to identify the predictors of residential mobility (Who moves?) in terms of individual-level characteristics including demographic factors, socioeconomic status (SES), and health status, as well as various environmental characteristics. Second, it aimed to characterise residential environment changes in terms of new relative to previous location factors for movers (Into which types of neighbourhoods do movers go?). Third, it aimed to assess, for movers, whether changes in features of environments differ according to household income (Does moving change the residential environment equally for all movers?). The fourth and last objective was to evaluate for cross-sectional estimations of place-health relationships the extent of bias associated with residential movements (In comparison to longitudinal analyses which account for residential mobility, do cross-sectional analyses in which mobility is unaccounted for provide reasonable interpretations of place effects on health?).

## 2. Materials and Method

### 2.1. Data source

Data from the household component of the National Population Health Study (NPHS), a longitudinal survey of the ten Canadian provinces initiated in 1994/1995, was used for analysis. The NPHS uses a closed cohort that was representative of the Canadian population 12 years and older in 1994/1995. The household component of the NPHS excludes persons living on Indian Reserves and Crown Lands, full-time members of the Canadian Forces, residents of health institutions, and residents of remote areas in provinces of Ontario and Quebec. Eight biennial follow-ups are available, yielding nine waves of observations from 1994/1995 to 2010/2011. Statistics Canada, which conducted the survey and disseminates data, sub-sampled individuals who provided a full response for all nine waves, or until death. From this sub-sample, individuals aged of 55 years and over in 1998/1999 were selected for analysis. Use of the third wave of the survey (1998/1999) as baseline for this analysis was justified as income data were collected categorically in the first two waves vis-à-vis others for which continuous income data were collected, thus enabling accounting for inflation (annualised income, see below). Adjustment for inflation was necessary as change in income was analysed in association with the odds of moving (see below). This study focuses on community-living individuals.

### 2.2. Variables

#### 2.2.1. Residential mobility

Discordance in a respondent's complete, six-digit postal code between any two consecutive waves was considered to reflect residential mobility. Any missing values for mobility reflected incomplete, missing or erroneous postal codes at either of the two consecutive waves for which mobility was assessed, otherwise, institutionalisation or death at the subsequent wave.

#### 2.2.2. Individual-level factors

Demographic data included gender, age group (55–59, 60–64, 65–69, 70–74, 75–79, 80–84, or 85 years and older), immigration status (Canadian-born, or not), living arrangement (alone, with spouse and children, with spouse only, with children and without spouse, or other arrangement), being a retiree (or not), and owning one's dwelling (or not). Socioeconomic status (SES) was assessed as individual educational attainment (less than secondary school graduation, secondary school graduation, some post-secondary education, or post-secondary graduation) and quintiles of annualised household income. Household income was annualised based on Statistics Canada's province-specific consumer price index and then categorised into quintiles for regional strata (four strata: Atlantic provinces; Quebec; Ontario; and western provinces). Health status was assessed as self-reported health (“In general, would you say your health is…”). Self-reported health has been shown to be a strong predictor of mortality for various age groups [12]–[14], and across socioeconomic groups [15]. It also predicts functional decline in older adults [13].

#### 2.2.3. Environmental factors

Neighbourhood features were expressed at the level of enumeration areas (EA) level and characterised according to rural-urban denomination, area-level SES, and housing quality. The EA is the smallest unit for which census data are available for the years covered by this analysis. EAs have average population of 650 individuals (SD = 373) across the ten-province sample. Respondents were geocoded to EAs based on their residential postal code.

Levels of urbanity/rurality were determined using a classification produced by Statistics Canada [16]. Municipalities were categorised as being part of either a census metropolitan area (CMA), a census agglomeration (CA), or as pertaining to the class of small cities and rural areas (i.e., not included in any CMA or CA). CMAs are groups of municipalities comprising more than 100,000 inhabitants. CAs have populations ranging from 10,000 to 100,000 inhabitants. Distinguishing the three larger cities (i.e., those with more than 1 million inhabitants) from other CMAs led to a four-level classification: (i) Toronto, Montreal or Vancouver CMAs, (ii) other CMAs, (iii) CAs, and (iv) small cities and rural areas. Individuals were assigned to these urbanity/rurality categories based on the municipality in which their residential EA was located.

Area-level SES was estimated using a deprivation index resulting from a principal component analysis involving six census variables: proportion of individuals (15 yrs and over) employed; proportion of individuals with a high school degree; average income; proportion of persons living alone; proportion of individuals separated, divorced or widowed; and proportion of single-parent household [17],[18]. The principal component analysis produced two factors labelled material deprivation (high loadings on employment, education and income variables) and social deprivation (high loadings on living alone, marital status and single-parenting variables).

Neighbourhood-level housing quality was operationalised as the proportion of individuals who declared that their dwelling needed major repairs (the higher this proportion, the lower the housing quality). The variable is believed to reflect the physical state of the built environment.

Variables reflecting material deprivation, social deprivation and housing quality variable were classified into population-weighted quintiles. Such quintiles were specific to regions (i.e., Atlantic provinces, Québec and Ontario individually, and western provinces).

### 2.3. Statistical Analyses

In order to identify predictors of residential mobility (first objective), multi-level logistic regression was used for modelling the odds of mobility at any given wave relative to the next wave. Periodic observations were treated as repeated measures (level 1) clustered within individuals (level 2) [19]. Gender and immigrant status were modelled at level 2 as time-invariant factors. All other variables were treated as time-varying factors and thus modelled at level 1. Measures of change were derived from the comparison of values between two consecutive waves: change in annualised income quintile, change in professional status (none, retiring, back to work), children leaving home, and separation or widowhood. Change measures were determined for the same period as residential mobility, that is, between a given wave and the next wave. Differences in the nature of residential environments among movers (second and third objectives) were assessed using descriptive statistics.

The impact of accounting or not accounting for residential mobility in estimating associations between residential environment characteristics and individual health (fourth objective) was assessed by comparing three multi-level logistic regressions in which self-assessed health was the outcome. In a first regression, environmental characteristics were modelled as time-varying characteristics (i.e., at level 1). In two other regressions, instead of allowing environmental characteristics to vary over time, the same value was repeated (at level 1) using (a) the baseline value and (b) the latest observed value. Data preparation and computation of descriptive statistics were performed using SAS, version 9.3 (SAS Institute, Cary, NC). Multilevel modelling was undertaken using HLM version 6.08 (Scientific Software International, Lincolnwood, IL). Weighting was used to support the representativeness of the sample analysed. Weights were produced by Statistics Canada and reflect the structure of the overall Canadian population in 1994/1995.

## 3. Results

The study sample comprised 2553 individuals aged 55 years and older in 1998/1999, following the exclusion of 352 individuals due to incomplete data on at least one individual-level variable (except household income for which a category for missing data was created due to the high proportion of missing information). Table 1 describes the sample prior to and following these exclusions. Although exclusion from the sample was related to all variables baring gender and urban/rural denomination, exclusions did not substantially modify the distribution of the observed data.

**Table 1.** Sample characteristics at baseline, Canadians of 55 years or older in 1998/99.

Characteristic		sample before exclusions		sample after exclusions		Comparison of excluded and non-excluded *	
		*n*	%	*n*	%	*χ*^2^	***P*-value**
Gender						2.89	0.089
Women		1578	54.3	1400	54.8		
Men		1327	45.7	1153	45.2		
Age						264.66	<0.001
55–59		631	21.7	598	23.4		
60–64		577	19.9	527	20.7		
65–69		549	18.9	498	19.5		
70–74		425	14.6	363	14.2		
75–79		367	12.6	319	12.5		
80–84		213	7.3	164	6.4		
85+		142	4.9	84	3.3		
Living arrangement						33.40	<0.001
with spouse only		1449	49.9	1290	50.5		
with spouse & children		373	12.8	347	13.6		
single-parenting		69	2.4	61	2.4		
alone		792	27.2	666	26.1		
other		223	7.7	188	7.4		
Education						19.05	<0.001
less than secondary school graduation		1223	42.1	1044	40.9		
secondary school graduation		382	13.1	336	13.2		
some post-secondary		601	20.7	542	21.2		
post-secondary graduation		699	24.1	631	24.7		
Household income						60.33	<0.001
Quintile 1 (lowest)		481	16.6	398	15.6		
Quintile 2		466	16.1	405	15.9		
Quintile 3		479	16.5	421	16.5		
Quintile 4		480	16.5	442	17.3		
Quintile 5 (highest)		412	14.2	386	15.1		
missing		586	20.2	501	19.6		
Immigrant						23.80	<0.001
Immigrant		554	19.1	489	19.2		
non immigrant		2349	80.9	2064	80.8		
missing		2	0.1				
Retired						55.59	<0.001
yes		1856	63.9	1592	62.3		
no		1032	35.5	961	37.7		
missing		17	0.6				
Dwelling owned by household member						28.35	<0.001
yes		2251	77.5	2009	78.7		
no		654	22.5	544	21.3		
Self-assessed health						95.07	<0.001
poor		526	18.1	409	16.0		
good		2379	81.9	2144	84.0		
Housing quality						84.31	<0.001
Quintile 1 (highest)		497	17.1	430	16.9		
Quintile 2		631	21.7	559	21.9		
Quintile 3		555	19.1	497	19.5		
Quintile 4		593	20.4	530	20.7		
Quintile 5 (lowest)		610	21.0	536	21.0		
missing		18	0.6				
Material deprivation						371.39	<0.001
Quintile 1 (lowest)		562	19.3	505	19.8		
Quintile 2		586	20.2	533	20.9		
Quintile 3		532	18.3	483	18.9		
Quintile 4		586	20.2	528	20.7		
Quintile 5 (highest)		580	20.0	504	19.7		
missing		58	2.0				
Social deprivation						371.84	<0.001
Quintile 1 (lowest)		540	18.6	494	19.4		
Quintile 2		513	17.7	455	17.8		
Quintile 3		558	19.2	504	19.7		
Quintile 4		586	20.2	519	20.3		
Quintile 5 (highest)		649	22.3	581	22.8		
missing		58	2.0				
Urban/Rural						9.41	0.052
Toronto, Montreal or Vancouver CMAs		587	20.2	502	19.6		
other CMAs		873	30.0	770	30.2		
CAs		573	19.7	511	20.0		
small cities and rural areas		871	30.0	770	30.1		
missing		2	0.1				

* A Chi-square test was used for assessing the relationship between each characteristic and the exclusion status (to be excluded or not) of individuals from the complete sample.

Among those 2553 individuals followed over the 12-year period, 1722 (67.5%) did not move (Table 2). Over the entire period, 1080 moves were observed. These moves were made by 831 different individuals, of whom 643 moved only once (representing 77.4% of movers and 25.2% of the study population).

**Table 2.** Frequency of residential mobility.

Nb. of moves	Individuals	
	*n*	%
0	1722	67.5
1	643	25.2
2	145	5.7
3	26	1.0
4 or 5	16	0.6

### 3.1. Predictors of mobility

The odds of moving did not vary according to gender or immigration status but was inversely associated with age (Table 3, model 1). Living arrangement was associated with moving: relative to individuals living with a spouse and without children, individuals living with a spouse and children were less likely to move while individuals living alone or in the ‘other’ category (i.e., living with a brother, a sister, parents or unrelated persons) were more likely to move. Change in living arrangement was associated with an effect of greater magnitude than was living arrangement *per se*: a child or children leaving the household, and separation or widowhood were each associated with strong differences in the odds of moving, relative to households for which no move occurred (ORs = 3.15 (95% CI: 1.78–5.57) and 2.54 (95% CI: 1.76–3.69), respectively).

Education was associated with residential mobility: relative to individuals with a post-secondary education, those having some post-secondary education (without graduation) had a higher odds of moving (OR = 1.27 (95% CI: 1.00–1.60)). Household income was not associated with moving. Substantial change in income level (i.e., decrease corresponding to 2 quintiles or more) was, however, associated with moving (OR = 1.85 (95% CI: 1.18–2.91)). Owning one's dwelling indicated lesser odds of moving (OR = 0.50 (95% CI: 0.40–0.62)). Further, whilst being a retiree was not associated with moving independent of other covariates, returning to work after having initially retired was associated with moving (OR = 1.89 (95% CI:1.34–2.67)).

No associations were observed across levels of housing quality, or for material deprivation or urban-rural categories. Social deprivation tended to be related to greater odds of moving (not all ORs were statistically significant), but the similarity of the observed differences suggests it is the reference group (lowest social deprivation) that is characterised by lowest odds of moving in comparison to all other levels of social deprivation.

Adjusting for self-assessed health did not meaningfully change the magnitude or interpretations of estimated associations between moving and the variables described above (Table 3, model 1 compared to model 2). Where changes were observed, variations were modest at best. Self-assessed poor relative to good health was associated with elevated odds of moving (OR = 1.36 (95% CI: 1.07–1.72)) while change in self-assessed health was not associated with moving.

**Table 3.** Predictors of residential mobility.

		Model 1		Model 2	
		OR	(95% IC)	OR	(95% IC)
Gender					
Women		*referent*		*referent*	
Men		0.96	(0.80–1.16)	0.96	(0.80–1.16)
Age group					
55–59		*referent*		*referent*	
60–64		0.73	(0.53–0.99)	0.72	(0.53–0.99)
65–69		0.56	(0.41–0.79)	0.56	(0.40–0.78)
70–74		0.43	(0.30–0.62)	0.42	(0.29–0.61)
75–79		0.55	(0.37–0.80)	0.53	(0.36–0.78)
80–84		0.61	(0.40–0.91)	0.58	(0.39–0.87)
85 +		0.51	(0.32–0.81)	0.49	(0.31–0.78)
Immigration status					
Canadian-born		*referent*		*referent*	
immigrant		0.86	(0.67–1.10)	0.84	(0.66–1.09)
Living arrangement					
with spouse only		*referent*		*referent*	
with spouse & children		0.62	(0.39–0.98)	0.60	(0.38–0.96)
single-parenting		1.09	(0.65–1.83)	1.08	(0.64–1.82)
alone		1.37	(1.09–1.72)	1.39	(1.10–1.74)
other		1.90	(1.37–2.63)	1.91	(1.38–2.64)
Change in living arrangement					
Children leaving		3.15	(1.78–5.57)	3.16	(1.79–5.59)
Widowhood/separation		2.54	(1.76–3.69)	2.57	(1.77–3.73)
Education					
less than secondary school graduation		0.90	(0.71–1.13)	0.87	(0.69–1.09)
secondary school graduation		0.85	(0.64–1.15)	0.84	(0.63–1.14)
some post-secondary		1.27	(1.00–1.60)	1.24	(0.99–1.57)
post-secondary graduation		*referent*		*referent*	
Household income					
Quintile 1 (lowest)		1.14	(0.79–1.67)	1.09	(0.75–1.60)
Quintile 2		0.88	(0.62–1.25)	0.86	(0.60–1.23)
Quintile 3		1.11	(0.80–1.55)	1.09	(0.78–1.52)
Quintile 4		1.04	(0.75–1.44)	1.04	(0.75–1.44)
Quintile 5 (highest)		*referent*		*referent*	
n/a		1.06	(0.75–1.50)	1.05	(0.75–1.49)
Change in income quintile					
none		*referent*		*referent*	
+1		1.00	(0.71–1.40)	1.00	(0.72–1.41)
+2 or more		1.19	(0.64–2.21)	1.24	(0.67–2.30)
−1		1.18	(0.87–1.60)	1.17	(0.86–1.59)
−2 or more		1.85	(1.18–2.91)	1.85	(1.18–2.91)
n/a		1.01	(0.81–1.27)	1.00	(0.80–1.25)
Owner		0.50	(0.40–0.62)	0.51	(0.41–0.64)
Retiree		1.26	(0.95–1.67)	1.28	(0.97–1.70)
Change in professional status					
none		*referent*		*referent*	
became a retiree		1.17	(0.84–1.65)	1.15	(0.82–1.61)
back to work		1.89	(1.34–2.67)	1.84	(1.30–2.59)
Housing quality					
Quintile 1 (highest)		*referent*		*referent*	
Quintile 2		0.82	(0.62–1.08)	0.81	(0.62–1.07)
Quintile 3		0.96	(0.73–1.26)	0.96	(0.73–1.25)
Quintile 4		0.99	(0.76–1.30)	0.98	(0.75–1.29)
Quintile 5 (lowest)		0.85	(0.64–1.13)	0.85	(0.63–1.13)
Material deprivation					
Quintile 1 (lowest)		*referent*		*referent*	
Quintile 2		0.90	(0.69–1.17)	0.89	(0.68–1.16)
Quintile 3		1.19	(0.92–1.56)	1.17	(0.90–1.53)
Quintile 4		1.20	(0.91–1.57)	1.17	(0.90–1.54)
Quintile 5 (highest)		1.11	(0.83–1.49)	1.08	(0.80–1.45)
Social deprivation					
Quintile 1 (lowest)		*referent*		*referent*	
Quintile 2		1.32	(0.99–1.77)	1.29	(0.97–1.73)
Quintile 3		1.36	(1.01–1.83)	1.35	(1.00–1.81)
Quintile 4		1.37	(1.02–1.82)	1.36	(1.02–1.81)
Quintile 5 (highest)		1.32	(0.98–1.80)	1.30	(0.96–1.76)
Urban/Rural					
Toronto, Montreal or Vancouver census metropolitan area (CMA)		*referent*		*referent*	
other CMAs		1.10	(0.85–1.42)	1.08	(0.83–1.39)
CAs		1.05	(0.80–1.39)	1.03	(0.78–1.36)
small cities and rural areas		0.92	(0.69–1.23)	0.92	(0.69–1.22)
Self-assessed health					
good				*referent*	
poor				1.36	(1.07–1.72)
Change in self-assessed health					
none				*referent*	
from good to poor				0.89	(0.67–1.18)
from poor to good				1.12	(0.81–1.57)

### 3.2. Changes in residential environments

Table 4 presents a cross-tabulation of *moves* (not movers) comparing origin (lines) and destination (columns) neighbourhoods. The cells of the diagonal contain moves for which the destination neighbourhood is categorised in the same quintile as the neighbourhood of origin. The lowest levels of housing quality show a low proportion of moves from and to neighbourhoods of essentially the same character. Moves occurring between neighbourhoods of different levels of housing quality (off-diagonal) represented a larger proportion of moves than those occurring within the same type of neighbourhood (diagonal). For moves occurring between neighbourhoods of different character (off-diagonal), there was no clear trend in the magnitude of change.

In terms of material deprivation, proportion of moves from and to neighbourhoods of the same quintile (Table 4, diagonal) tended to be inversely associated with deprivation, although the relationship was not monotonic. For social deprivation, the relationship appeared to be J-shaped: the lowest proportions were observed for the second and third quintiles whilst the first and fourth quintiles had higher, similar proportions with the highest probability as observed for the fifth quintile (Table 4, diagonal). For both types of deprivation, no clear trends were observed in terms of proportion of moves from and to neighbourhoods of different levels of deprivation (Table 4, off-diagonals).

In terms of urban-rural differences, Table 4 shows that 85.2% of movers residing in a CMA moved to a place located in a CMA. A majority of moves originating from a CA or from rural areas were made towards a similar environment (52.8% for moves from a CA and 58.5% for moves from a rural area). Moves occurring from a CA to another type of environment were proportionately more numerous for moves to a rural area than to a CMA (this difference may not be meaningful given small numbers). Moves from a rural area to outside a rural area were dominantly made to a CA.

Looking at the distribution of *movers* (not moves) in terms of origin-destination differences (i.e., change in quintile in new versus former neighbourhood), Table 5 shows that overall, for roughly one third of movers, moving did not lead to a change in type of neighbourhood (housing quality: 29.7%; material deprivation: 35.6%; and social deprivation: 30.7%). The proportion of those for whom the level of housing quality worsened (28.6%) was slightly lower than that for whom it improved (36.8%). The opposite was observed for material deprivation (worsening: 32.0%; and improvement: 24.8%) but not for social deprivation (worsening: 27.5%; and improvement: 34.2%). When analysed across levels of household income, origin-destination differences were only statistically different for social deprivation (*χ*^2^ = 28.39, *p* = 0.002, Table 5), but no obvious trend was observed. This association was weaker (*χ*^2^ = 15.95, *p* = 0.043) when origin-destination differences and household income effects were estimated when excluding individuals with missing data on income.

### 3.3. Accounting for mobility in estimating place-health associations

Table 6 presents associations between neighbourhood characteristics and poor self-assessed health estimated from models which differ in how residential mobility is accounted for or not accounted for. No statistically significant differences in self-assessed health were observed across levels of housing quality, when residential mobility was accounted for (Table 6, model 1). When residential mobility was not accounted for (Table 6, models 2 and 3), a significant difference in self-assessed health was observed for the lowest level of housing quality. Also, not accounting for residential mobility led, in general, to greater differences in self-assessed health than was the case for differences observed when accounting for residential mobility.

**Table 4.** Distribution of neighbourhood characteristics, before and after moving, for all observed moves (*n* = 1080).

Before moving (origin)			After moving (destination)								Chi-square tests **	
			Quintile 1 (lowest)	Quintile 2		Quintile 3	Quintile 4	Quintile 5 (highest)		n/a *	*χ*^2^	*p*
Housing quality											86.77	<0.001
Quintile 1 (highest)		*n*	**53**	34		33	25	20		26		
		row %	**27.5%**	17.9%		17.3%	13.2%	10.5%		13.7%		
Quintile 2		*n*	31	**72**		42	35	16		23		
		row %	14.3%	**32.8%**		19.1%	15.7%	7.4%		10.7%		
Quintile 3		*n*	32	38		**60**	28	32		32		
		row %	14.4%	16.9%		**26.9%**	12.7%	14.6%		14.4%		
Quintile 4		*n*	45	40		28	**50**	37		36		
		row %	19.0%	16.9%		12.0%	**21.1%**	15.6%		15.4%		
Quintile 5 (lowest)		*n*	32	36		24	57	**41**		18		
		row %	15.3%	17.1%		11.7%	27.3%	**19.9%**		8.7%		
Material deprivation											208.00	<0.001
Quintile 1 (lowest)		*n*	**80**	36		32	17	15		36		
		row %	**36.9%**	16.5%		14.9%	8.1%	6.9%		16.7%		
Quintile 2		*n*	42	**61**		25	27	20		36		
		row %	19.7%	**29.0%**		11.7%	13.0%	9.5%		17.2%		
Quintile 3		*n*	27	48		**78**	28	18		26		
		row %	12.1%	21.2%		**34.8%**	12.6%	8.0%		11.3%		
Quintile 4		*n*	27	35		36	**62**	41		30		
		row %	11.7%	15.3%		15.6%	**26.7%**	17.6%		13.1%		
Quintile 5 (highest)		*n*	21	14		28	47	**53**		33		
		row %	10.8%	7.0%		14.3%	24.0%	**27.0%**		16.9%		
Social deprivation											142.33	<0.001
Quintile 1 (lowest)		*n*	**42**	18		31	24	15		18		
		row %	**28.1%**	12.1%		21.1%	16.3%	10.2%		12.2%		
Quintile 2		*n*	20	**39**		28	38	34		26		
		row %	10.8%	**21.2%**		15.3%	20.8%	18.2%		13.9%		
Quintile 3		*n*	24	33		**42**	40	34		33		
		row %	11.5%	16.2%		**20.5%**	19.4%	16.5%		15.9%		
Quintile 4		*n*	19	23		24	**69**	67		40		
		row %	7.9%	9.5%		9.7%	**28.6%**	27.8%		16.4%		
Quintile 5 (highest)		*n*	20	33		31	45	**125**		45		
		row %	6.5%	11.1%		10.2%	15.2%	**41.8%**		15.2%		
			**After moving (destination)**									
**Before moving (origin)**			CMA ***		CA		small city / rural		n/a *			
Urban/Rural											812.01	<0.001
CMA ***		*n*	**445**		24		16		37			
		row %	**85.2%**		4.6%		3.1%		7.1%			
CA		*n*	25		**150**		41		68			
		row %	8.9%		**52.8%**		14.3%		24.1%			
small city / rural		*n*	38		47		**160**		28			
		row %	13.8%		17.4%		**58.5%**		10.3%			

* The nature of the destination neighbourhood was not available either because the postal code could not be associated with an enumeration area or because the environmental variable was missing for the enumeration area.

** Tests on the association between change between origin and destination types of neighbourhood. Tests were made using contingency tables for which missing values were excluded.

*** Data for CMAs of Toronto, Montreal and Vancouver were grouped with those for other CMAs as Statistics Canada's dissemination rules do not allow for the dissemination of small frequencies for ensuring confidentiality of surveys' respondents. When separating the CMAs of Toronto, Montreal and Vancouver from other CMAs, some frequencies were considered too small to be disseminated.

**Table 5.** Change in neighbourhood characteristics among movers (*n* = 831) *.

Change (in quintiles)		total	by household income						Chi-square tests**	
			Quintile 1 (lowest)	Quintile 2	Quintile 3	Quintile 4	Quintile 5 (highest)	n/a	*χ*^2^	*p*
**Housing quality**									13.32	0.206
decrease by ≥1 (improvement)	*n*	305	55	49	58	54	39	50		
	col %	36.8%	40.8%	34.8%	46.3%	39.1%	33.1%	28.8%		
0 (no change)	*n*	247	41	44	34	47	32	49		
	col %	29.7%	30.0%	31.1%	26.6%	34.1%	27.4%	28.7%		
increase by ≥1 (worsening)	*n*	238	36	42	34	31	44	51		
	col %	28.6%	26.9%	29.9%	26.6%	22.1%	37.2%	29.7%		
n/a ***	*n*	41	3	6	1	7	3	22		
	col %	4.9%	2.3%	4.2%	0.5%	4.7%	2.3%	12.8%		
**Material deprivation**									8.69	0.562
decrease by ≥1 (worsening)	*n*	266	46	50	44	37	43	46		
	col %	32.0%	33.6%	35.4%	34.9%	27.0%	36.6%	26.6%		
0 (no change)	*n*	296	46	48	42	53	41	66		
	col %	35.6%	34.2%	33.9%	33.0%	38.4%	34.7%	38.2%		
increase by ≥1 (improvement)	*n*	206	36	32	35	40	28	37		
	col %	24.8%	26.3%	22.5%	27.5%	28.7%	23.7%	21.3%		
n/a ***	*n*	63	8	12	6	8	6	24		
	col %	7.6%	5.9%	8.2%	4.6%	5.9%	5.0%	13.9%		
**Social deprivation**									28.39	0.002
decrease by ≥1 (worsening)	*n*	228	45	35	32	34	42	41		
	col %	27.5%	33.2%	24.6%	25.3%	24.5%	35.7%	23.6%		
0 (no change)	*n*	255	47	53	29	44	25	57		
	col %	30.7%	34.7%	37.8%	23.0%	31.9%	21.2%	32.9%		
increase by ≥1 (improvement)	*n*	284	36	41	59	52	45	51		
	col %	34.2%	26.2%	29.4%	47.1%	37.7%	38.1%	29.6%		
n/a ***	*n*	63	8	12	6	8	6	24		
	col %	7.6%	5.9%	8.2%	4.6%	5.9%	5.0%	13.9%		
**TOTAL**		831	136	141	126	138	118	172		

* For those individuals who moved more than once (n = 187), average difference is reported.

** Tests on the association between change on residential type and household income. Tests were made using contingency tables for which missing values were excluded.

*** The nature of the destination neighbourhood was not available because either the postal code could not be associated with an enumeration area or because the environmental variable was missing for the enumeration area.

For material deprivation, the odds of poor self-assessed health associated with increasing deprivation rose across the three models, the greatest differences observed when accounting for mobility (model 1). Change in the odds of poor health associated with rising social deprivation were non-monotonic, with differences of small magnitude between the estimated effects for any given quintile across the three models.

In terms of urban-rural denominations, no statistically significant differences were observed in the odds of poor self-assessed health in all the three models shown in Table 6. Differences across the three models were modest, at best, with the exception of observed differences between the largest metropolitan areas (Toronto, Montreal or Vancouver) and other CMAs or the CAs which were weaker for model 3 than for the two other models.

**Table 6.** Associations between neighbourhood characteristics and poor self-assessed health *.

		Model 1 ^†^		Model 2 ^‡^		Model 3 ^§^	
		OR	(95% IC)	OR	(95% IC)	OR	(95% IC)
Housing quality							
Quintile 1 (highest)							
Quintile 2		1.15	(0.85–1.57)	1.38	(0.99–1.93)	1.22	(0.88–1.69)
Quintile 3		1.10	(0.81–1.50)	1.36	(0.96–1.88)	1.37	(0.99–1.91)
Quintile 4		1.10	(0.81–1.49)	1.28	(0.91–1.79)	1.32	(0.95–1.84)
Quintile 5 (lowest)		1.15	(0.84–1.58)	1.43	(1.01–2.02)	1.42	(1.01–1.99)
Material deprivation							
Quintile 1 (lowest)							
Quintile 2		1.34	(1.00–1.79)	1.25	(0.92–1.72)	1.40	(1.03–1.92)
Quintile 3		1.42	(1.05–1.93)	1.45	(1.04–2.00)	1.37	(0.98–1.90)
Quintile 4		1.60	(1.18–2.17)	1.53	(1.11–2.12)	1.58	(1.14–2.20)
Quintile 5 (highest)		1.79	(1.29–2.48)	1.63	(1.15–2.32)	1.65	(1.16–2.33)
Social deprivation							
Quintile 1 (lowest)							
Quintile 2		1.45	(1.06–1.98)	1.51	(1.09–2.10)	1.37	(0.99–1.90)
Quintile 3		1.20	(0.88–1.64)	1.22	(0.88–1.70)	1.18	(0.85–1.64)
Quintile 4		1.17	(0.86–1.60)	1.40	(1.01–1.95)	1.05	(0.76–1.45)
Quintile 5 (highest)		1.54	(1.12–2.11)	1.61	(1.15–2.26)	1.68	(1.21–2.34)
Urban/Rural							
Toronto, Montreal or Vancouver census metropolitan area (CMA)							
other CMAs		1.30	(0.96–1.77)	1.35	(0.99–1.83)	1.23	(0.90–1.67)
CAs		1.25	(0.91–1.73)	1.25	(0.90–1.74)	1.14	(0.82–1.59)
small cities and rural areas		0.97	(0.71–1.34)	1.00	(0.72–1.39)	0.99	(0.71–1.38)

* Accounting for gender, age group, living arrangement, education, and household income.

† Environmental characteristics are modelled as time-varying characteristics, allowing for changes in characteristics following residential mobility.

‡ Baseline environmental characteristics are repeated for all observations (no changes throughout follow-up).

§ The last observed environmental characteristics are repeated for all observations (no changes throughout follow-up).

## 4. Discussion

Of the four objectives of this study, the first was to identify demographic, social and health factors associated with moving among older Canadians. The remaining objectives were to describe changes in the type of neighbourhood experienced by movers and evaluate if such changes were associated with household income. This study also sought to determine whether the use of cross-sectional data for estimating place effects on health leads to bias on the basis of exposure misclassification arising from residential mobility.

Our results are consistent with the life-course perspective on residential mobility which posits that residential mobility is greatly influenced by age-related events [7],[8]. We found for individuals aged 55 years and older that younger age, children taking their own residence, separation or widowhood, returning to work after having retired, not owning one's dwelling, and experiencing an important reduction in income were each statistically significant predictors of moving. Two age-related events were not observed, however, to be associated with moving: retirement, and declining health. Being a retiree and retiring are not predictors of moving independent of other predictors of late-life mobility. This finding is likely to be an indication of the absence of effect of retirement and not an artefact of statistical over-adjustment (colinearity) for other age-related events coinciding with retirement. In effect, for models estimating the association between retirement and moving while accounting for age and gender alone (data not shown), the estimated coefficients and their standard errors were similar to those presented in Table 3. It could be that in our study population, retirement influences mobility through changes in income level or through anticipation (i.e., pre-retirement mobility) following changes in living arrangement (children leaving, widowhood or separation).

Poor self-assessed health at baseline predicted residential mobility in our sample. That health status predicts residential mobility is likely to be of particular importance for decision-makers, because residential mobility has potential to affect the health profile of local populations, or at least of service seekers. Indeed, previous research has shown that health problems may lead to residential mobility as individuals seek to improve their access to support and health-related services [6],[7]. This behaviour could explain the higher prevalence of disability in central places where services are clustered [20].

During the 16-year observation period of this study, 3 out of 10 Canadians aged 55 years or older moved at least once. The majority of these moves led to a change in the neighbourhood status of the mover (measured in quintiles). These changes were not limited to similar type of neighbourhood (e.g., from 2nd to 3rd quintile or from 5th to 4th quintile). Changes of large magnitude (e.g., >2 quintiles) were observed in non-negligible proportions. Further, residential mobility led to positive as well as negative changes in neighbourhood status: one third of all individuals moved to a neighbourhood of lower status, another third moved to a neighbourhood of higher status, and the remaining third moves to a neighbourhood of similar status. This distribution holds for housing quality as well as for material and social deprivation. These results demonstrate that among older Canadians, over approximately one and a half decades, movers can be exposed to multiple types of neighbourhoods.

Changes in neighbourhood types due to moving were not related to (i.e., not conditioned by) household income when neighbourhood changes were analysed across levels of housing quality and material deprivation. However, changes across levels of social deprivation had a statistically significant association with household income. Our study indicates that movers from low-income households (quintiles 1 and 2) were less likely to experience improvement in neighbourhood social deprivation. However, aside from this observation, there was no clear, general trend observed in the statistically significant association between household income and changes in movers' level of neighbourhood social deprivation. Not accounting individuals with unknown income, the latter association was weaker and at the limit of statistical significance. Combined with the fact that education and household income were not associated with moving, these results have important implications for distinguishing between the compositional and contextual origins of the spatial patterning of health in multilevel analyses.

Multilevel regression models used for estimating the associations between health and contextual factors are commonly built adopting a two-stage approach, adjusting for individual-level predictors of health status, for example SES, prior to the inclusion of contextual factors [21]. Such adjustment is justified if individual-level SES is a potential confounder of the health-context association, but it is *not* justified if individual-level SES is an intervening variable in the pathway of the contextual effect on local populations' health [22]. In the latter situation, controlling for individual-level SES can lead to biased estimations of the health-context association, through over-adjustment. Our results suggest that, at least among older adults, individual-level SES is neither a predictor of moving nor of its consequences in terms of type of neighbourhood. Thus, an analytical strategy for estimating place effects on health while accounting for individual-level SES should involve mediation analyses, which allow for differentiating direct and indirect effects.

This study aimed also at evaluating whether estimations of place effects on health made using cross-sectional data were influenced by exposure misclassification due to residential mobility. Longitudinal data were used to assess place and health associations, allowing for residential mobility. These estimations were used as the referent for estimating bias (misclassification) associated with use of cross-sectional data. Cross-sectional analyses were undertaken for two series of models which used the first and last observations for environmental characteristics pertaining to movers. These results suggest that there are no systematic biases in estimating place effects on health using cross-sectional data. In effect, estimates are relatively stable for social and material deprivation whereas for housing quality, using cross-sectional data may lead to over-estimation of differences between extremes levels. Thus, even if misclassification does occur, its effect on the health-context association is generally small.

The above small impact of not accounting for residential mobility was observed despite some notable variations in the nature of neighbourhood features experienced for movers. This could be attributable to different factors, potentially acting together. First, there was no structure observed for movers in the nature of changes in residential area exposures. Thus, any effect of variations in residential area exposures over time may be averaged out. Second, the association between place and health may be driven by longer induction period than that applicable here, or even by an induction period having occurred prior to our study period. Hence, our analysis would not be adequately sensitive to the impact of residential mobility. Third, movers experiencing change in the nature of their neighbourhood may simply be too few by which to evidence an association (32.5% of the population are movers and roughly one third of movers experience such a change).

This study has limitations. One is our operationalisation of residential mobility on the basis of differences between residential postal codes. Postal codes are the most precise geographic locator available from the NPHS. For urban areas in Canada, quite unlike the United States, postal codes pertain to very small territories (approximately one side of one street block). Moves occurring within a single postal code territory are not likely to occur often. In rural areas, however, postal codes are associated with larger territories and it is thus there is a greater likelihood that moves could have occurred without being captured by our study. The proportion of such moves is impossible to estimate using the available data and, to our knowledge, no previous study can shed light on this issue. Consequently, there is a potential for under-estimation of moves amongst rural inhabitants in our study. This could have led to an overestimation of the changes in the nature of neighbourhoods (moves occurring in rural areas being more likely to occur between residential environments of similar nature given the socioeconomic homogeneity of rural areas, relative to urban areas). Another limitation is the frequency of the measures (NPHS waves). Changes in individual-level health status over time are not necessarily part of a linear progression from perfect to bad health. Transitions can occur as rapidly as monthly [23],[24]. Brief episodes of health changes (i.e., <2 years) would not have been captured by the survey protocol used in this study. It is possible that any such changes experienced by individuals led to residential mobility. Missing short-term transitions as these may have led to an underestimation of the effect of health changes on the odds of moving, but the importance of this bias is unknown.

Our study indicates that health status not individual-level SES is associated with moving. It indicates furthermore that residential mobility is associated with change in type of neighbourhood for a majority of movers and that residential mobility leads to a fairly large variability in the magnitude of such changes. However, changes in residential area exposures among movers do not appear to impact the estimation of place effects on health where based on analysis of cross-sectional data. Our analyses do not allow for assessing the impact of mobility on variation in the population-level association between SES and health over time. However, this study suggests that residential mobility among older adults may not be an important factor explaining secular changes in social inequalities in health among older adults. This is because, as shown, moving was not associated with individual-level SES or with most contextual factors. Also, this study suggests that estimating place effects on health while controlling for the “independent” effect of individual-level SES may lead to biased estimations on the basis that individual-level SES may not be a confounder, but rather, an intermediate factor. There is a shortage of studies assessing how residential mobility can lead to biases estimations of place effect on health using cross-sectional data. Health and place research would benefit from further studies analysing potential bias attributable to residential mobility and in particular studies assessing if such a bias is sensitive to age groups being analysed.
